# Art therapy with children surviving cancer used to relieve symptoms associated with death, loss and pain

**DOI:** 10.1192/j.eurpsy.2021.1809

**Published:** 2021-08-13

**Authors:** C. Emilia

**Affiliations:** Mental Health Center For Children And Adolescents. Stationary Day Neurology And Pediatric Psychiatry., Emergency Clinic Hospital for Children, Cluj-Napoca, România, Cluj-Napoca, Romania

**Keywords:** dying is inevitable, emotional disorders, non-verbal communication, art therapy an resilience in per

## Abstract

**Introduction:**

**Figure:**
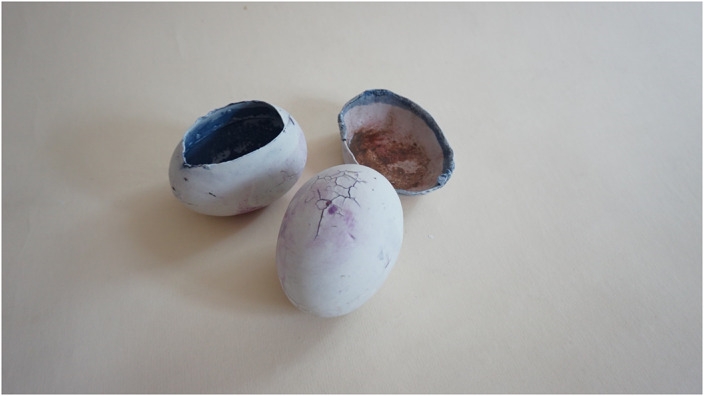

Since dying is inevitable, it is part of life, children need to be able to deal with the feelings and emotions associated withs death, loss and pain. When the grieven child move among the art modalities, he or she is able to deepen understaning of his or her lived experiences.

**Objectives:**

**Figure:**
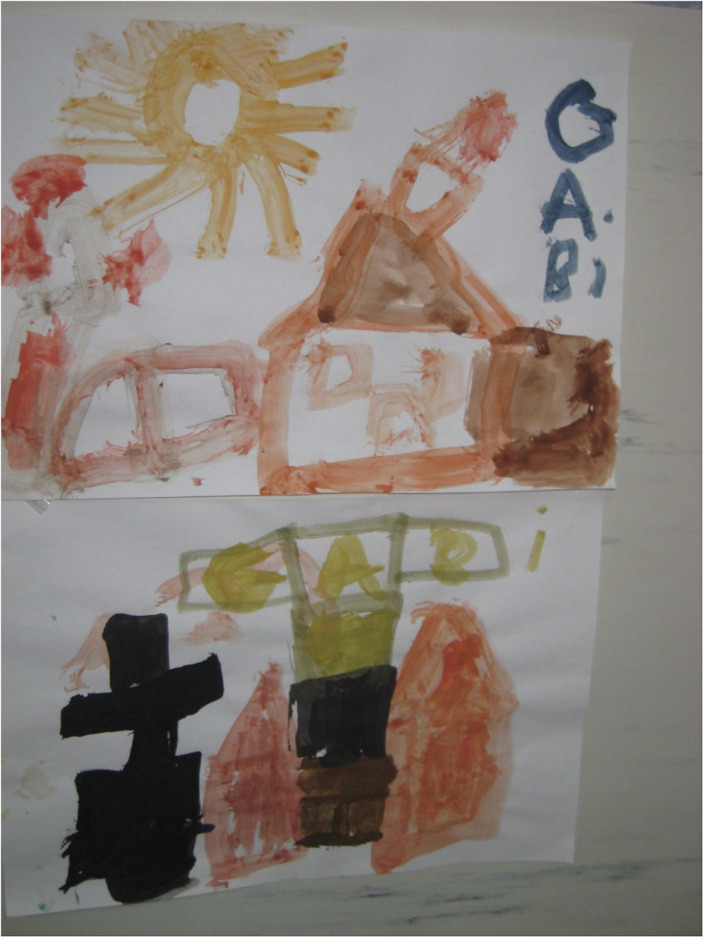

Our aim is to uncover these new perspectives and sources of inspiration in order to advance in defining the importance of resilience in personal development.

**Methods:**

We made use of the following techniques: ceramic, drawing, modeling, painting, assemblage of unconventional materials, multimedia techniques, animation. Performing artworks, artefacts, or using craft arts are test activities for art therapy and occupational therapy. „…Contemporary visual arts bring together, in different degrees of relationship and fusion, fields of art that until now were understood and practiced more individually. The most suitable territory for this partnership is that of the physical and metaphysical environment, provided by the installationist and shareholder arts.” [2] A medical project was transformed into an artistic project [4]

**Figure:**
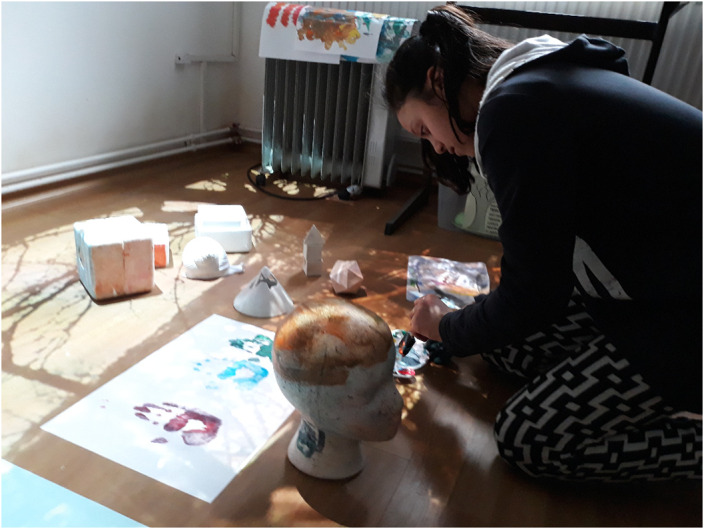

**Results:**

Given the diversity of non-verbal communication of the child, art therapy is not a simple accessory method in the therapeutic process of emotional disorders caused by grief of children, but a mandatory condition of it.

**Conclusions:**

Given the diversity of non-verbal communication of the child, art therapy is not a simple accessory method in the therapeutic process of emotional disorders caused by grief of children, but a mandatory condition of it.

**Disclosure:**

REFERENCES [1] Drăgan-Chirilă, Diana.(24-26.05 2018), Associate Professor Ph.D., University of Art and Design Cluj-Napoca, Romania, visual artist, Coordinator of the multimedia installation “Diagnostique” new media and multimedia performance instal

